# Defining low-risk group before surgical treatment in endometrial cancer: A retrospective review

**DOI:** 10.1097/MD.0000000000049188

**Published:** 2026-06-19

**Authors:** Alpaslan Kaban, Alp Koray Kinter, Emine Ufuk Büyükkaya Öcal, Ali Kasapoğlu, Aydin Kilinç, Teksin Polat, Yunus İlhan

**Affiliations:** a Gynecological Oncology Department, Istanbul Prof. Dr. Cemil Taşcioğlu City Hospital, Istanbul, Turkey; b Department of Pathology, Istanbul Prof. Dr. Cemil Taşcioğlu City Hospital, Istanbul, Turkey.

**Keywords:** endometrial neoplasms, hysterectomy, lymph node excision, salpingo-oophorectomy

## Abstract

Endometrial cancer is a heterogeneous malignancy involving many criteria considered in treatment planning. This study analyzed risk factors associated with postoperative outcomes in 250 women diagnosed with endometrioid-type endometrial adenocarcinoma. The aim of this analysis was to identify the subgroup with the lowest risk of extrauterine metastasis, which could be exempted from comprehensive staging surgery. Criteria for admission to the study were endometrioid histology, grade 1 or 2 tumors, and tumor invasion of half the myometrium based on pathology reports. Extrauterine metastases, such as lymph node, fallopian tube, ovary, and omentum involvement, were evaluated. Of the 250 patients, 178 (71.2%) had grade 1 tumors and 72 (28.8%) grade 2 tumors; 152 (60.8%) had a tumor size of 3 cm or less and 98 (39.2%) had a tumor size of more than 3 cm. Eighteen patients (7.2%) had extrauterine metastases. Patients with grade 2 tumors had a higher rate of extrauterine metastases compared with those with grade 1 tumors (15.3% vs 3.9%, *P* = .003). Patients with tumor size >3 cm had a higher rate of extrauterine metastases compared with those with tumor size 3 cm or less (13.3% vs 3.3%, *P* = .006). In multivariate analysis, grade 2 (hazard ratio = 3.66, 95% confidence interval: 1.33–10.08; *P* = .012) and tumor size >3 cm (hazard ratio = 3.83, 95% confidence interval: 1.28–11.39; *P* = .016) were independent factors associated with extrauterine metastases. According to the findings of this study, among patients with endometrial cancer with <50% myometrial invasion depth, those with grade 1 tumors and tumor diameters smaller than 3 cm had the lowest rate of extrauterine metastasis. Tumor size was identified as a potential factor in determining the risk of extrauterine metastasis. These results support avoiding comprehensive staging surgery in the subgroup meeting the criteria of having a tumor smaller than 3 cm and a grade 1 endometrioid tumor.

## 1. Introduction

Endometrial cancer (EC) is the most common gynecologic malignancy in developed countries and its prevalence is increasing.^[1,2]^ This cancer is a malignancy that arises in the epithelial lining of the uterus.^[3]^ Therefore, irregular bleeding or postmenopausal bleeding is the most common symptom of this disease, and it is diagnosed by biopsy of the endometrial tissue.^[4–6]^ Historically, EC has been classified into 2 main clinicopathological and molecular types: type I is the much more common endometrioid adenocarcinoma (80%–90%) and type II comprises non-endometrioid subtypes, such as serous, clear-cell, and undifferentiated carcinomas, as well as carcinosarcoma/malignant-mixed Müllerian tumor (10%–20%).^[7]^

The International Federation of Gynecology and Obstetrics (FIGO) changed the staging of EC from clinical to surgical-based staging in 1988 and further detailed this staging in 2023.^[8]^ According to this staging system, disease limited to the uterus is roughly stage I or II, while the presence of extrauterine metastases means that the disease is stage III or IV.^[8]^ According to FIGO, the standard treatment for patients with EC is comprehensive staging surgery that includes total hysterectomy, bilateral salpingo-oophorectomy, pelvic, and para-aortic lymph node dissection. Omentectomy, peritoneal biopsies, and peritoneal washings are less routinely performed.^[9–11]^ Whether comprehensive staging surgery is necessary for all patients with EC remains an open question. Comprehensive staging surgery carries risks such as prolonging the duration of the surgery, vascular injury, bleeding, transfusion, lymphocele or lymphedema formation, and also requires a more specialized surgeon.^[11–14]^ Indeed, in the context of improving patient outcomes and management of healthcare resources, identifying low-risk groups is crucial and it makes sense to avoid extensive staging surgery in this patient group. In addition, randomized controlled trials have not shown evidence that comprehensive staging surgery, including lymphadenectomy in women with presumed stage I disease, reduces the risk of death or disease recurrence.^[14,15]^ A systematic review and meta-analysis of randomized controlled trials of routine adjuvant radiotherapy to treat possible lymph node metastases in women with early-stage EC did not find a survival advantage.^[16]^ On the other hand, in a study conducted by the Gynecologic Oncology Group, approximately 22% of patients with clinical stage I EC who underwent comprehensive surgical staging were found to have metastatic disease and 11% to have lymph node metastases.^[17]^ It seems necessary to perform subgroup analyses and plan comprehensive staging surgery according to risk groups in clinical stage I disease. Avoiding unnecessary comprehensive staging surgery in low-risk groups is an appropriate approach. European Society for Medical Oncology (ESMO), European Society for Radiotherapy and Oncology (ESTRO), and European Society of Gynaecological Oncology (ESGO) Endometrial Consensus Conference Working Group published a report on this subject, stating that patients with grade 1 or 2 endometrioid-type tumors with a myometrial invasion rate of 50% or less are defined as the low-risk group.^[7]^ This report did not specify tumor size as a prognostic factor. Although tumor size is not a criterion of the FIGO staging system, many studies have shown a significant association between tumor size and extrauterine metastasis.^[18–22]^

Preoperative risk stratification and appropriate surgical staging based on this stratification in patients with EC are essential to reduce patient morbidity and ensure the efficient use of physician and healthcare resources. Many studies have been conducted on risk-stratification-based treatment. The Korean Gynecologic Oncology Group recommended serum CA-125 levels and 3 parameters (deep myometrial invasion, lymph node enlargement, and extension beyond the uterine corpus) to define the low-risk group before surgery.^[23]^ Han et al, in their study of 300 patients, found that in addition to these criteria, being over 55 years of age was also a risk criterion for extrauterine metastasis.^[24]^ According to the Mayo Criteria, low-risk patients for extrauterine metastasis were reported as having <50% invasion, tumor size <2 cm, and grade 1 or grade 2 endometrioid histology.^[25]^ Bauer et al suggested that tumor size of 5 cm and criteria for myometrial invasion are the best predictors of extrauterine metastasis.^[26]^ Accumulating evidence suggests that tumor size has the potential to be a prognostic criterion for EC treatment. Recently, it is advised to also subdivide EC into 4 molecular subgroups. Each subgroup is highly associated with a certain risk of recurrence and helps to decide on adjuvant treatment.^[10]^

Risk criteria for EC are not yet clearly defined. These predictive criteria for extrauterine metastasis are important for clinicians in determining surgical treatment. Identifying a low-risk group helps avoid complications of unnecessary surgery and prevents wasted healthcare costs. Therefore, we planned a single-center retrospective study to evaluate risk factors such as tumor grade, tumor size, and myometrial invasion associated with postoperative outcomes in 250 women with EC.

## 2. Methods

### 2.1. Patient selection

**Figure 1.**
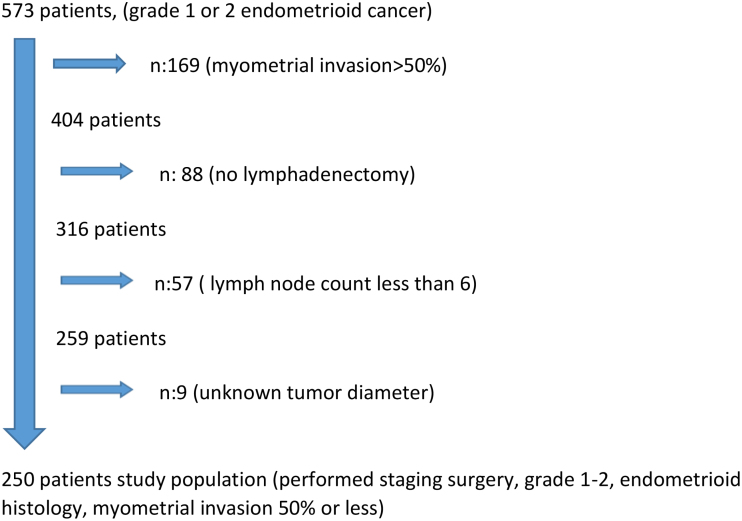
Study flowchart.

**Figure 2.**
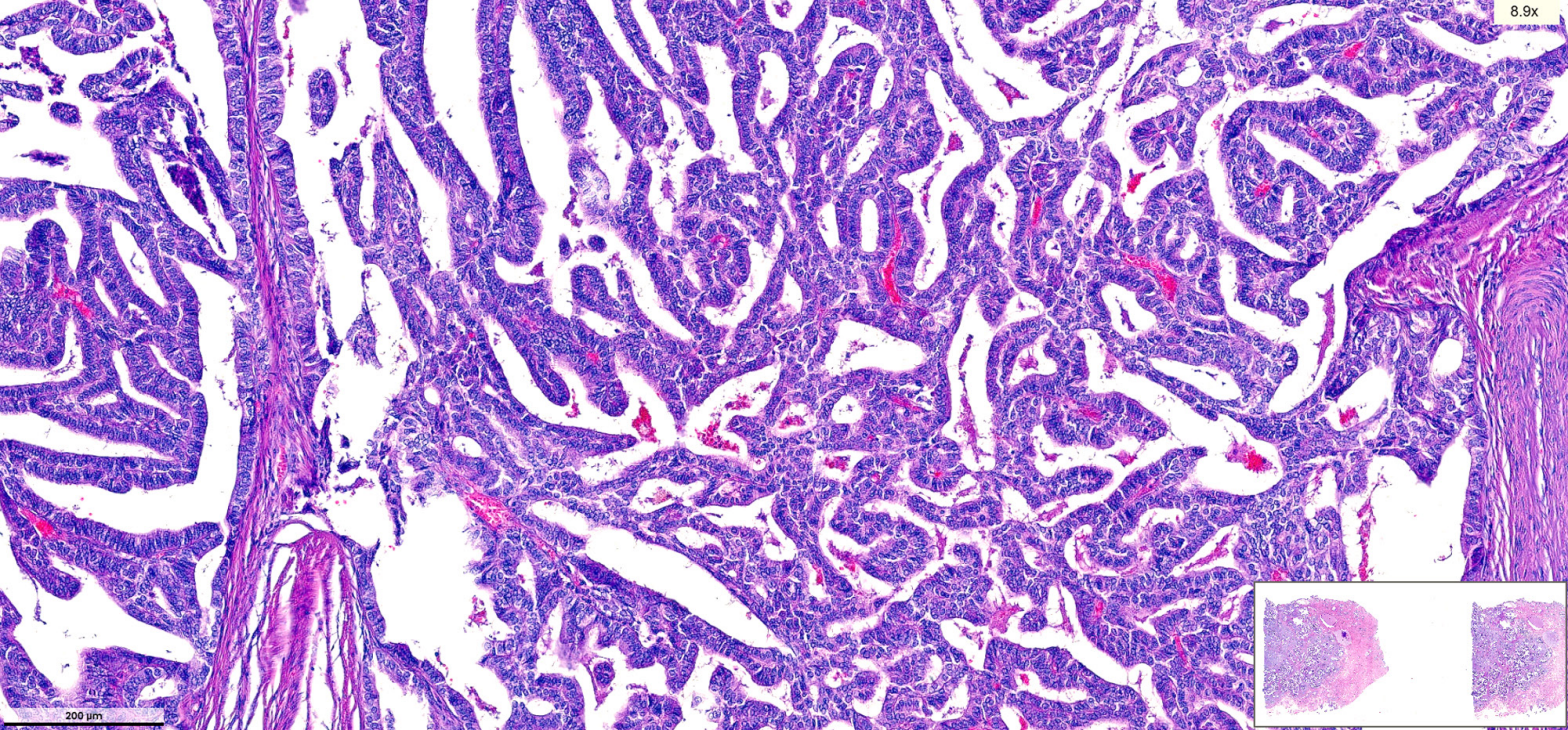
Pathological preparation image of grade 1 endometrioid adenocarcinoma.

**Figure 3.**
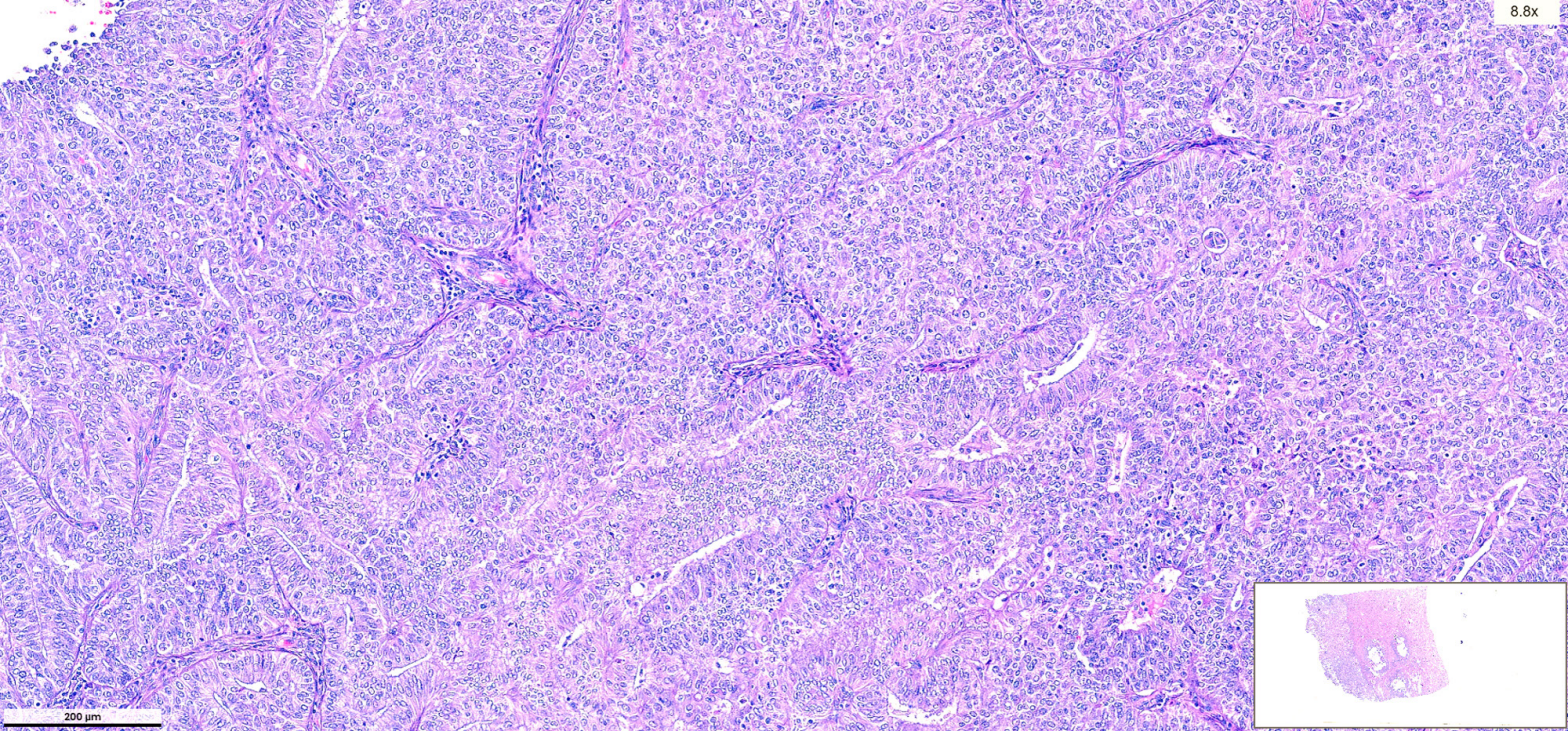
Pathological preparation image of grade 2 endometrioid adenocarcinoma.

In this study, the records of 573 EC patients who underwent surgery between January 2004 and December 2024 were retrospectively reviewed. The inclusion criteria for the study were as follows: endometrioid-type histology, histological grade 1 or 2, <50% myometrial invasion, lymphadenectomy (pelvic ± para-aortic, at least 6 lymph nodes). The study flowchart is presented in Figure 1. The following patients did not meet the inclusion criteria and were excluded from the study: 88 patients who did not undergo lymphadenectomy; 57 patients who underwent lymphadenectomy but had fewer than 6 lymph nodes removed; 169 patients with more than 50% myometrial invasion; and 9 patients with unknown tumor diameter. Data from patients excluded from the study were not included in the analyses. This study analyzed 250 patients who underwent comprehensive staging surgery. The comprehensive staging surgery included hysterectomy, bilateral salpingo-oophorectomy, peritoneal lavage, and pelvic ± para-aortic lymphadenectomy procedures. Preoperative evaluations of patients undergoing surgery were mostly performed using magnetic resonance imaging and transvaginal ultrasound. The criteria evaluated were myometrial invasion depth, tumor size, and tumor location. Most patients underwent open surgery. A small percentage (~10%) underwent laparoscopic surgery. For pelvic lymphadenectomy, fatty lymphatic tissues around the external iliac vessels and in the obturator fossa were removed. For para-aortic lymphadenectomy, fatty tissue around the aorta and inferior vena cava (mostly to the inferior mesenteric artery, sometimes up to the left renal vein) were removed. Since the patients were in the low-risk group, lymph node sampling was preferred over aggressive lymphadenectomy. The maximum number of lymph nodes removed was 48. Tumor size was the largest value of the 3-dimensional measurements measured by gynecopathologists and reported in the final pathology report. Clinical and pathological characteristics of the patients, including age, histological subtype, tumor grade, depth of myometrial invasion, tumor size, number of lymph nodes, lymph node metastasis, and extrauterine metastases, were recorded. Extrauterine metastases were considered to include pelvic ± para-aortic nodal metastasis, adnexal metastasis, omental metastasis, and other pelvic tissue, if present. Tumor grade was determined under a microscope by the pathologist according to the proportion of solid areas in a tissue sample of the uterine endometrium that was surgically resected: low grade = grade 1 (≤5%) and grade 2 (6%–50%); high grade = grade 3 (>50%). Excessive nuclear atypia increases the grade of a grade 1 or 2 tumor by 1 (Figs. 2 and 3). The FIGO 2023 EC staging criteria were used for staging in the study. Accordingly, a tumor limited to the uterine corpus is defined as stage I. If there is no myometrial invasion, it is defined as IA; if present, it is defined as IB.^[8]^

This study received approval number 100 from the local ethics committee on April 5, 2024.

### 2.2. Statistical analysis

The Statistical Package for the Social Sciences (SPSS) for Windows version 21 (IBM Corporation, 2012) was used to perform all analyses. Numerical data are expressed in terms of number of cases (percentage, n) for descriptive values in the study cohort. Group comparisons with and without extrauterine metastasis were conducted using the chi-square test or Fisher method. The Mann–Whitney *U* test was used to analyze the difference in lymph node counts. First, univariate analysis was performed to screen for extrauterine metastases, evaluating age, number of lymph nodes removed, tumor grade, and tumor size. Then, multivariate logistic regression analysis was performed, including age, tumor size, and tumor grade to screen for independent risk factors and eliminate confounding factors. The difference was considered statistically significant at *P* < .05.

## 3. Results

**Figure 4.**
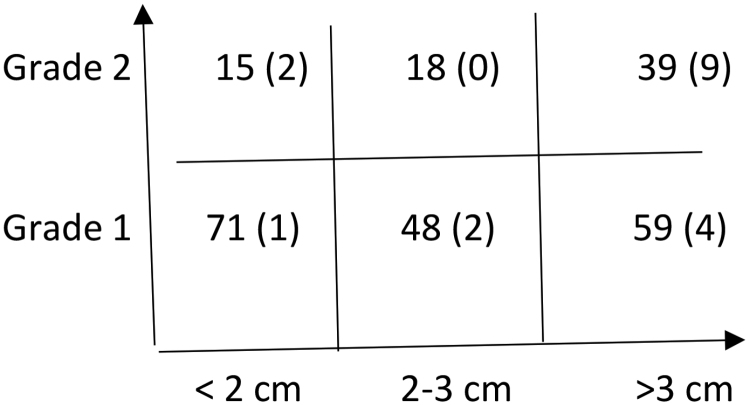
Distribution of patients according to grade and tumor size (the number in parentheses indicates the number of patients with metastases).

A total of 250 patients with a median age of 56 years (range 26 to 87) were analyzed. Surgicopathological characteristics of patients are presented in Table 1. The distribution of patients according to grade and tumor size is presented in Figure 4. Extrauterine metastases were found in 18 (7.2%) patients. Nodal metastasis was found in 6 patients, adnexal metastasis in 14 patients, omental metastasis in 1 patient (some patients had multiple metastases). Of the 250 patients, 98 (39.2%) had a tumor diameter >3 cm and 152 (60.8%) had a tumor size of 3 cm or less. One hundred seventy-eight patients (71.2%) had grade 1 tumors and 72 patients (28.8%) had grade 2 tumors.

**Table 1:** Characteristics of 250 patients with low-risk endometrial cancer.

Characteristics	Value	%
Age, yr		
Median	56 (26–87)	
<60	173	68.9
>60	72	31.4
Removed lymph node count		
Median (min–max)	13 (6–48)	
Mean ± SD	14.94 ± 8.2	
Extrauterine disease		
Yes	18	7.2
No	232	92.8
Distributions of extrauterine metastasis*		
Nodal metastasis	6	2.4
Adnexal met	14	5.6
Omental metastasis	1	<1
Malignant cells in peritoneal washings	4	1.6
Tumor grade 1	178	71.2
Tumor grade 2	72	28.8
Tumor diameter		
≤2 cm	86	34.4
2–3 cm	66	26.4
>3 cm	98	39.2
Extrauterine met/grade 1 and tumor size of ≤2 cm	1/71	1.4
Extrauterine met/grade 1 and tumor size of 2–3 cm	2/48	4.1
Extrauterine met/grade 1 and tumor size of >3 cm	4/59	6.8
Extrauterine met/grade 2 and tumor size of ≤2 cm	2/15	13
Extrauterine met/grade 2 and tumor size of 2–3 cm	0/0	–
Extrauterine met/grade 2 and tumor size of >3 cm	9/39	23.1

max = maximum, met = metastasis, min = minimum, SD = standard deviation.

* Some patients had multiple metastases.

### 3.1. Univariate analyses

The tumor grade and the tumor size were found to be significant predictors of extrauterine disease in univariate analysis. Patients with tumors larger than 3 cm had a higher rate of extrauterine metastasis than patients with smaller tumors (13.3% vs 3.3%, *P* value = .006, odds ratio [OR] 4.49, 95% confidence interval [CI]: 1.54–13.04). Similarly, patients with grade 2 tumors had a higher rate of extrauterine metastasis than patients with grade 1 tumors (15.3% vs 3.9%, *P* = .003, OR 4.40, 95% CI: 1.63–11.8; Table 2).

**Table 2:** Characteristics of 250 patients stratified by extrauterine involvement.

	Extrauterine metastasis		*P* value	HR (95% CI)
	Yes (N = 18)	No (N = 232)		
Age, yr				
Mean ± SD	56.1 ± 9.4	56.0 ± 9.8	.966	–
≤60	14	159		
>60	4	73		
Removed lymph nodes count, (median)	17 (8–48)	13 (6–44)	.504	–
Grade 1	7 (3.9)	171 (96.1)	**.003**	4.40 (1.63–11.8)
Grade 2	11 (15.3)	61 (84.7)		
Tumor diameter (cm)				
≤3	5 (3.3)	147 (96.7)	**.006**	4.49 (1.54–13.04)
>3	13 (13.3)	85 (86.7)		

The values in bold are statistically significant.

CI = confidence interval, cm = centimeter, HR = hazard ratio, SD = standard deviation.

### 3.2. Multivariate analyses

Multivariate analysis was performed to determine the independent factors associated with extrauterine metastasis. The variables included in the multivariate analysis were age, tumor size, and tumor grade. According to the multivariate analysis, tumor size and tumor grade were found to be independent factors for extrauterine metastasis, but age was not (*P* = .016, OR 3.83, 95% CI: 1.28–11.39; *P* = .012, OR 3.66, 95% CI: 1.33–10.06; Table 3).

**Table 3:** Results of multivariate analyses of odds ratios in the logistic regression model with extrauterine disease as the dependent variable.

	Reference	Multivariate analysis		
		*P* value	OR	95% CI
Age	–	.759	0.992	0.944–1.043
Tumor size	≤3 cm	**.016**	3.830	1.287–11.395
Grade	1	**.012**	3.664	1.331–10.086
Constant		.022	0.033	

The values in bold are statistically significant.

CI = confidence interval, OR = odds ratio.

## 4. Discussion

In this study, the extrauterine metastasis rates of EC cases with grade 1 or 2 endometrioid type and less than half myometrial invasion were examined. The extrauterine metastasis rate was found to be 7.2% in this group of patients with EC. The question our study attempted to answer was whether we could identify new subgroups with statistically significant differences in risk (either lower or higher) by performing subgroup analyses. Accordingly, we calculated that the rate of extrauterine metastasis was significantly lower in patients with grade 1 tumors smaller than 3 cm. Metastasis was detected in 3 of 119 patients in this subgroup (2.5%). The rate of extrauterine metastasis in the group with grade 2 tumors larger than 3 cm was found to be 23.1% (Table 1). Multivariate analysis showed that grade 2 tumors or tumors larger than 3 cm were independent risk factors (Table 3). According to this result, in patients with endometrioid-type EC with myometrial invasion, grade 2 tumor or tumor larger than 3 cm may be criteria for recommending comprehensive staging surgery.

Studies suggest that comprehensive staging surgery can be avoided in the low-risk group in patients with EC. Comprehensive staging surgery is a procedure associated with morbidities such as bleeding, transfusion, vascular injury, lymphocyst, lymphatic edema, and longer surgery; it also requires a specialist surgeon. It is advantageous to identify risk groups in EC patients and to avoid aggressive surgery in low-risk groups. However, there is no complete consensus on the criteria to be included to identify a low-risk group. EC cases meeting the criteria for grade 1 or 2 endometrioid type and less than half myometrial invasion have been identified as low-risk groups in many studies.^[7,13–15,20]^ Our study did not confirm that grade 2 tumors are low-risk group criteria.

In 1987, the Gynecological Oncology Group stratified patients with EC into low, moderate, or high risk groups based on the degree and depth of myometrial invasion.^[17]^ The authors did not assess the primary tumor size as a risk factor. In our study, we identified the potential of tumor size to determine risk groups. Firstly, Schink et al showed that tumor size is an important predictor of lymphatic involvement in EC.^[18]^ In their study, they found that the rate of nodal metastasis was 4% among patients with a tumor size of ≤2 cm and 15% among those with a tumor size of >2 cm. In our study, the cutoff value for tumor size was 3 cm. The rate of metastasis was 3% in the group <3 cm and 13% in those >3 cm in our study. This value was statistically significant. Mariani et al. published a study that included 328 patients with grade 1 or 2 endometrioid EC. The authors described patients with endometrioid histology, <2 cm tumor diameter, grade 1 or 2, and <50% myometrial invasion as a low-risk group for extrauterine metastases (Mayo Criteria) and indicated that lymphadenectomy was unnecessary for these patients and that hysterectomy with bilateral salpingo-oophorectomy is sufficient.^[19]^ When we adapted the present study to the Mayo criteria (Table 4), the rate of avoiding aggressive surgery was 34.4%, compared with 47.6% with our proposed criteria. These criteria of the present study spare more patients from aggressive surgery.

**Table 4:** Definition of the low-risk group, that may not require comprehensive surgery according to Mayo Criteria, ESMO-ESGO-ESTRO recommendations, and the results of the present study.

	According to ESMO-ESGO-ESTRO working group	According to Mayo Criteria	Present study
Low-risk criteria	Grade 1 or 2 and myometrial invasion < 50%	Grade 1 or 2, myometrial invasion < 50%, and tumor size of ≤2 cm	Grade 1, myometrial invasion < 50%, and tumor size of ≤3 cm
Not recommended for comprehensive staging surgery, % (n)	100% (250)	34.4% (86)	47.6% (119)
Extrauterine metastasis rate	7.2% (18/250)	3.5% (3/86)	2.5% (3/119)

ESGO = European Society of Gynaecological Oncology, ESMO = European Society for Medical Oncology, ESTRO = European Society for Radiotherapy and Oncology.

In the Surveillance, Epidemiology, and End Results Program analysis by Vargas et al., patients with histological grade 1 or 2, myometrial invasion <50%, and tumor size smaller than 2 cm were defined as the low-risk group, and the lymph node metastasis rate was found to be 1.4%.^[22]^ In these studies, patients with grade 2 tumors were also included in the low-risk group, and lymphadenectomy was deemed unnecessary in these patients. Unlike in our study, grade 2 tumors were defined as the risk group. In the present study, the rate of extrauterine metastases in patients with grade 2 tumors was found to be significantly higher than in patients with grade 1 tumors (15.3% vs 3.9%, *P* = .003). The presence of grade 2 tumors was found to be an independent risk factor for extrauterine metastases (OR 3.66, 95% CI: 1.33–10.08, *P* = .012; Table 3). In our study, patients with grade 2 tumors were not considered a low-risk group for extrauterine metastases. Tumor size was another factor evaluated to predict extrauterine metastases in the present study. Tumor size, which has prognostic potential in many reports, is not a component of the FIGO staging system.^[26–30]^ The ESMO-ESGO-ESTRO Endometrial Consensus Conference Working Group did not specify tumor size as a criterion for defining the risk groups.^[7]^ In some studies, a tumor size of 2 cm or less was included as a low-risk disease criterion.^[3,17,23–26]^ Berretta et al reported that patients who had grade 1 or 2 endometrioid uterine cancer, myometrial invasion < 50%, and a maximum tumor diameter of ≤3 cm can be treated with hysterectomy alone.^[29]^ In their studies, they pointed out that a tumor size of <3 cm is a low-risk criterion, in accordance with our study. In another study, Ytre-Hauge et al reported that a tumor diameter >4 cm was associated with the prediction of lymph node metastases.^[30]^ In our study, a tumor size of more than 3 cm was found to be an independent risk factor for extrauterine metastases (OR 3.83, 95% CI: 1.28–11.39, *P* = .016; Table 3). In many previous studies, patients with tumors >2 cm in size were defined as high-risk; therefore, lymphadenectomy was recommended in these patients.^[31]^ (We chose a 3-cm cutoff for tumor size because this cutoff value was statistically significant. When we analyzed using a 2-cm cutoff, there was no significant difference between the groups). In our opinion, unnecessary lymphadenectomy rates may increase if a 2 cm tumor size threshold is accepted for a comprehensive staging surgery decision.

The proportions of patients in the study cohort who did not require extensive staging surgery according to the ESMO-ESGO-ESTRO recommendations, Mayo Criteria, and the present study are presented in Table 4. When we compared the study cohort according to the 3 criteria, the rates of extrauterine metastasis were found to be the lowest in the present study (7.2%, 3.5%, and 2.5%, respectively). These rates indicate the rate of occult metastasis in the group exempted from comprehensive surgery.

The criteria for surgical planning should be those that can be determined before surgery. This study proposes risk stratification based on tumor grade, depth of invasion, and tumor size. These criteria can be obtained from preoperative evaluations. Tumor grade is determined by preoperative endometrial biopsy, and tumor size can be determined by imaging tests such as transvaginal ultrasound, magnetic resonance imaging, or intraoperative examination.^[32]^

The study has various limitations, including its retrospective nature, a limited number of cases, a lack of pathological slide reexamination, and differences in the number of resected lymph nodes. The results of the study need to be supported by larger-volume studies. The strengths of the study include the clear definition of inclusion criteria, the limited patient group, the fact that all cases were completely staged, and the fact that it was conducted by the same team in a single center.

## 5. Conclusion

The molecular and chemical factors that play a role in cancer development continue to be investigated worldwide.^[33–40]^ Meanwhile, studies to standardize treatment protocols are also ongoing.^[41]^ In this study, we aimed to identify a low-risk group of patients with EC. Avoiding unnecessary extensive surgery in this group is crucial for patient outcomes and the management of healthcare resources. According to the results of this study, patients with grade 1 endometrioid cancer, <50% myometrial invasion, and tumor diameter ≤3 cm can be treated with hysterectomy alone. The results of this study are encouraging in avoiding comprehensive staging surgery in patients with these criteria.

## Author contributions

**Conceptualization:** Alpaslan Kaban, Teksin Polat.

**Methodology:** Alpaslan Kaban, Aydin Kilinç.

**Data curation:** Alp Koray Kinter, Emine Ufuk Büyükkaya Öcal, Ali Kasapoğlu, Aydin Kilinç, Yunus İlhan.

**Formal analysis:** Alp Koray Kinter, Emine Ufuk Büyükkaya Öcal, Ali Kasapoğlu, Yunus İlhan.

**Supervision:** Alpaslan Kaban.

**Resources:** Alp Koray Kinter, Emine Ufuk Büyükkaya Öcal.

**Software:** Alp Koray Kinter.

**Visualization:** Alp Koray Kinter, Emine Ufuk Büyükkaya Öcal, Ali Kasapoğlu, Aydin Kilinç, Teksin Polat.

**Validation:** Emine Ufuk Büyükkaya Öcal, Ali Kasapoğlu, Aydin Kilinç.

**Investigation:** Yunus İlhan.

**Writing—original draft:** Alpaslan Kaban, Alp Koray Kinter, Emine Ufuk Büyükkaya Öcal, Ali Kasapoğlu, Aydin Kilinç.

**Writing—review & editing:** Alpaslan Kaban, Alp Koray Kinter, Emine Ufuk Büyükkaya Öcal, Ali Kasapoğlu, Aydin Kilinç.
